# Design of Glider Airborne Wind Turbine

**DOI:** 10.1155/tswj/8814936

**Published:** 2025-12-05

**Authors:** Salih N. Akour, Tareq Al-Soud, Rami Al-Balbeisi, Ali Al-Kabneh, Wesam S. Akour

**Affiliations:** ^1^ Mechanical Engineering Department, School of Engineering, The University of Jordan, Amman, Jordan, ju.edu.jo

## Abstract

Producing clean and renewable energy is the aim of many countries worldwide. Wind is one of the most vast renewable energy sources. High‐quality wind is available at high altitudes. To harvest such energy, wind turbines should reach such high altitudes. An airborne wind turbine system is conceptually designed to harvest wind energy at relatively high altitudes regardless of location. A glider is designed to carry a small wind turbine mounted at its nose. The glider is connected to the ground through a tether and electric wires to transmit power from the flying generator to the ground station. The resulting model airplane has a square wing with a Selig high‐lift, low‐Reynolds‐number airfoil section (S1223‐il) and a wingspan of 2 m. Tail airfoil sections are NASA airfoil 0012. The total mass of the glider is 3.35 kg. The aerodynamic design analysis is performed through CFD simulation. The forces and loads obtained from the CFD analysis are transferred to finite element software to perform structural analysis. Overshooting in lift and drag forces occurs in both cruise and nose‐up flights. Such overshoot behavior is eliminated by the wind turbine rotation effect. The developed model meets the design objectives successfully, since both structural and CFD analyses show the aircraft′s capability to carry the load. The CFD results prove that the glider is stable when the center of gravity is forward, and stability is achieved within 0.2 s. When the wind turbine is installed, there is slight oscillation in the lift force, but stability is reached within the design target of 0.2 s.

## 1. Introduction

Airborne wind energy (AWE) is the technology of generating energy using an airborne machine that transforms the kinetic energy of wind at high altitudes into electrical energy. Airborne wind energy systems (AWESs) fly in the air while connected by a tether and electrical cable to the ground, like kites or air balloons, rather than tower‐based systems that are fixed in position and altitude. Thus, to exploit the relative velocity of the air relative to the ground, AWESs are mechanically connected to the ground using tethers that provide anchorage against wind motion. Usually, tethers are connected to a ground station. Compared to tower‐based wind turbines, AWESs can collect wind power from high altitudes. It is well known that winds at higher altitudes are stronger and more consistent than those reachable by tower‐based systems [1–8].

AWESs have fewer limits on installation locations and capture wind energy at significantly high altitudes—up to 10 km above the ground. One of the basic principles was introduced by Loyd, who theoretically analyzed the maximum energy that can be extracted using an AWES′s tethered wing [9]. The maximum kinetic power that can be extracted from the wind near the ground surface using a tower‐based wind turbine is around 400 TW, compared to 1800 TW for an AWES operating in the higher atmospheric layers [10].

AWESs typically consist of two main parts: a ground unit and a flying unit that are connected mechanically and electrically to the ground unit using tethers and electrical cables. AWES falls into two main categories: ground‐based power generation systems (Ground‐Gen) and on‐board power generation systems (Fly‐Gen) [11]. In Fly‐Gen AWES, electrical energy is produced on board the aircraft using electrical generators equipped with wind turbines. The generated power is transmitted to the ground unit through electrical cables.

Compared to other AWES in terms of design and operation, glider AWES uses rigid wings that fly at high altitudes to maximize power generation. It generates power on‐board (via turbines on the glider), as seen in current designs like Makani′s rigid‐wing drones. Kite‐based systems—soft kites (e.g., SkySails) or hybrid designs (e.g., Skypull′s drone‐kite)—rely on aerodynamic lift and tether traction. They are typically Ground‐Gen, where the kite′s pull spins a ground‐based generator via a winch system. Aerostat (lighter‐than‐air) systems use helium‐filled balloons (e.g., Altaeros BAT) to lift turbines to high altitudes. They combine buoyancy and aerodynamic lift, often with fixed‐position turbines. Regarding efficiency and power output compared to other AWES, glider AWES offers high efficiency by leveraging steady high‐altitude winds and can achieve 5× the power of ground turbines with the same swept area. Kite systems are simpler but less efficient in turbulent winds; SkySails′ systems produce ~30 kW. Aerostats, such as Altaeros′ BAT, double the output of comparable ground turbines but are limited by balloon size and gas leakage [12–17].

This work documents the conceptual and preliminary design of a glider aircraft (often referred to as a glider) for a Fly‐Gen AWES. The research includes computational fluid dynamics (CFD) analysis and structural analysis using finite element analysis (FEA). Five different flight cases are investigated: a cruise case with the aerodynamic center ahead of the center of mass, a cruise case with the center of mass ahead of the aerodynamic center, a nose‐up case, a nose‐down case, and a cruise case with a wind turbine installed at the aircraft nose.

The novelty of this work lies in designing a glider capable of harvesting wind energy at high altitudes using a small wind turbine mounted at its nose. The glider is connected to the ground via a tether and electric cables, ensuring effective load carrying. The research demonstrates the glider′s effectiveness within a Fly‐Gen AWES, highlighting its desired performance, light weight, and strength, and offering a simplified design guideline. Furthermore, this flying generator enables harvesting from low wind speed regions at ground level by utilizing the high wind speed available at high altitudes.

## 2. Methods

The Fly‐Gen AWES consists of the following main components: the glider, the generator, and the tether that provides mechanical support and linkage to the ground unit. Understanding how aircraft work is crucial to achieving the desired aerodynamic design characteristics. Poorly defined aircraft invariably lead to suboptimal solutions.

The purpose of the glider in the Fly‐Gen AWES system is to carry the generator, the turbine, and the subcomponents of the system to a certain altitude. The main criterion for the glider is to be a high lifter at low wind speed, which corresponds to a relatively low Reynolds (Re) number.

The methodology followed to achieve the design is as follows:

1. A flying generator type is selected, and the lifter is a glider.
2. The wing airfoil is selected for its ability to generate high lift at low air speeds.
3. Symmetric airfoils are utilized for both the tail horizontal and vertical stabilizers.
4. Wing location should be determined. The high‐wing configuration is chosen because it is more stable and provides higher lift compared to other configurations.
5. Wing design parameters and sizing are determined, for example, wing cube load (WCL), aspect ratio (AR), lift and drag forces, and wing span.
6. Tail and body (fuselage) sizing are determined.
7. Geometry modeling is performed using a CAD software package.
8. CFD analysis is carried out to evaluate the lift and drag forces and to ensure stability.
9. Compare the lift forces obtained from CFD with those obtained from the analytical analysis.
10. Apply the CFD lift and drag forces to the structural model.
11. Conduct structural analysis using an appropriate CAD software package. The materials to be utilized in the physical model should be used to model the glider structure.
12. Analyze the results obtained from both models (CFD and structural) to ensure successful design achievement.

### 2.1. Wind Speed Variation With Altitude

**Figure 1:**
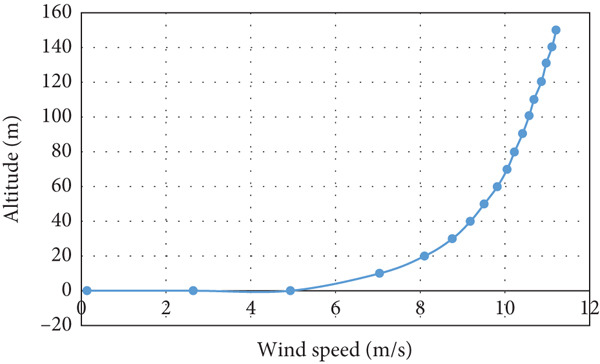
The wind speed variation with altitude according to Equation (1).

For a speed of 6 m/s at a height of 5 m above the ground and a surface roughness of 0.1, Figure 1 illustrates the variation of the wind speed profile with altitude calculated based on the logarithmic wind profile stated by Equation (1) [18]. It is evident that at an altitude of 20 m above the ground, the wind speed reaches approximately 8 m/s.

(1) v21=v∙lnh2/z0h1/z0

### 2.2. Design Strategy

The glider is assumed to have a total weight of 3.35 kg according to the total weight estimation that is carried in Appendices A, B and C. This means that the required lift force should be greater than 32.86 N. The wing and the tail assembly are the most important components in the design process. The following subsections provide details of the design parameters for these components.

#### 2.2.1. Wing Design

The primary function of the wing is to generate lift force. The wing is the main component of any aircraft for achieving lift capability and stability. It is responsible for carrying the aerodynamic and static loads.

The rectangular wing is suitable for small gliders with short wingspans. This type of wing is widely utilized in trainer gliders due to its simplicity in construction. It is a justifiable choice since the glider is a low‐speed and lightweight aircraft.

The WCL is defined as the total weight of the glider divided by the wing surface area (or the total lifting surface area if other surfaces generate lift force), as shown in Equation (2) [19]:

(2) WCL=WtotalAs1.5

Aircraft with WCLs greater than 12 generally have trouble flying, hard take‐off, and landing, and are unstable during maneuvering. For gliders, the best performance is when WCL is laying between 3.5 and 5.5 [20]. *A*_*s*_ is the wing surface area that can be calculated using Equation (3):

(3) As=Lw×Cm

where *L*_*w*_ is the wingspan in meter and *C*_*m*_ is the wing mean chord in meter.

The wing AR is defined by Equation (4) [20]:

(4) AR=Lw2As

Increasing the AR diminishes the induced drag and, therefore, increases efficiency. However, with equal wing spans, when increasing the AR, the wing area is reduced; then, the lift force generated is reduced as well. So the wing loading is increased. The acceptable range for the AR is 8 through 10.

The lift force generated by the wing is calculated using Equation (5):

(5) Lift force FLCLρ2AsVw2 and drag force FDCDρ2AsVw2

where *ρ* is the air density and *V*_*w*_ is the velocity of the glider, but here it represents the velocity of the wind flows relative to it, where the glider is fixed. Therefore, the minimum wind velocity needs is calculated by Equation (6):

(6) Vw=2 FLρ CL As or Vw=2 g Wtotalρ CL As

##### 2.2.1.1. Airfoil Selection

The airfoil section is the second most important wing parameter, after the wing planform area. The airfoil section is responsible for the generation of the optimum pressure distribution on the top and bottom surfaces of the wing such that the required lift force is generated with the lowest aerodynamic cost. To meet the goals, the selection criteria are as follows:

1. The airfoil of the wing should belong to the category of high‐lift, low‐speed airfoils.
2. The selected airfoil must have a maximum lift coefficient *C*_*L*_.
3. The selected airfoil must have a minimum drag coefficient *C*_*D*_.
4. The airfoil should have the highest lift curve slope.
5. The airfoil should have the highest lift‐to‐drag ratio.
6. The airfoil should have the lowest pitching moment coefficient *C*_*m*_.
7. The airfoil should have proper stall characteristics in the stall region (the lift loss should be gentle, not sharp).
8. The airfoil can be structurally reinforced. It should not be too thin for spars to be placed inside, and the cross section should be suitable for manufacturing as well.

The efficiency of the wing *E*_*f*_ is defined by Equation (7) as follows [21]:

(7) Ef=CLCD

**Figure 2:**
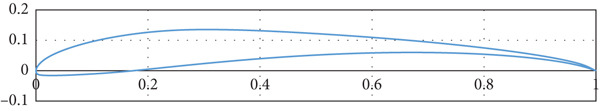
(S1223‐il) Selig high‐lift low‐Reynolds‐number airfoil section [22].

The airfoil is chosen to be S1223‐il, as illustrated in Figure 2: a Selig high‐lift, low‐Re‐number airfoil. The airfoil meets all the above criteria. The lift and drag characteristics of the S1223‐il shown in Figure 2 are presented in Figure 3. The lift coefficient is presented in Figure 3a for Re numbers of 100,000 and 200,000, which correspond to air speeds of 6 and 12 m/s, respectively. The drag coefficient, lift‐to‐drag ratio, and pitching moment coefficient are presented in Figures 3b, 3c, and 3d, respectively. The aircraft enters stall when the angle of attack exceeds 12° for a Re number of 200,000 and when it exceeds 10° for a Re number of 100,000.

Figure 3S1223‐il airfoil characteristics *Y* with the change in angle of attack alpha *X* at different values of Reynolds number [22]. (a) Left coefficient *C*_*L*_. (b) Drag coefficient *C*_*D*_. (c) *C*_*L*_/*C*_*D*_. (d) Pitching moment coefficient *C*_*m*_.

**Figure:**
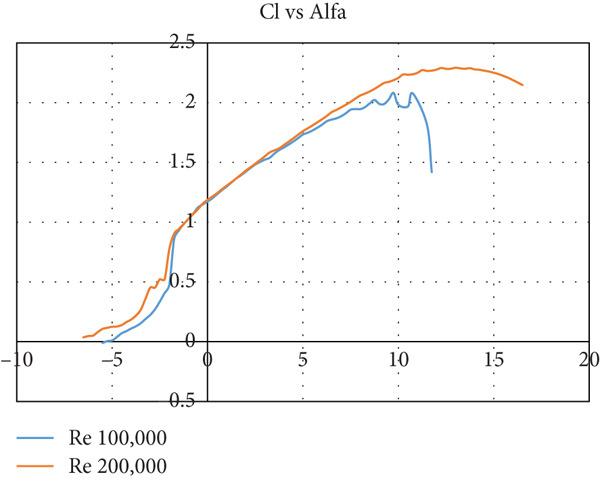
(a)

**Figure:**
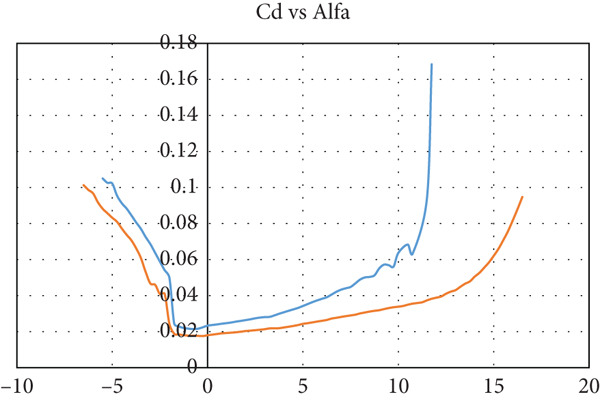
(b)

**Figure:**
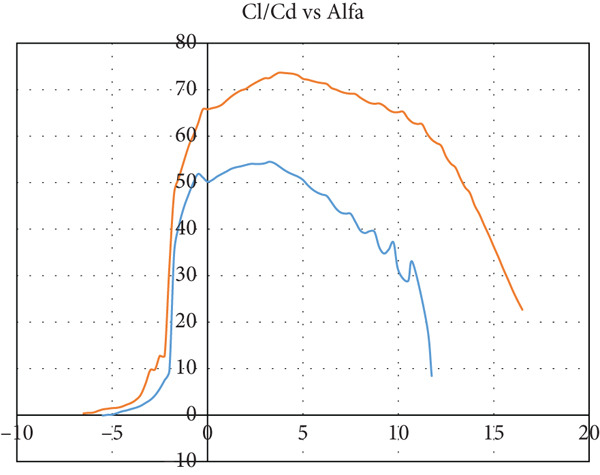
(c)

**Figure:**
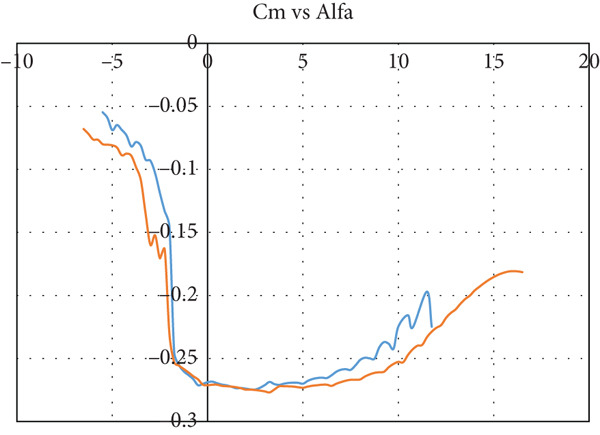
(d)

##### 2.2.1.2. Wing Vertical Location

One of the wing parameters that can be determined early in the wing design process is the wing′s vertical location relative to the fuselage centerline. This parameter directly influences the design of other aircraft components, including the aircraft tail design, landing gear design, and center of gravity (CG).

In principle, there are four options for the vertical location of the wing: (A) high wing, (B) mid wing, (C) low wing, and (D) parasol wing. The high wing configuration is chosen because it has several advantages that make it suitable for use, such as the following [21]:

1. It is more stable since the aircraft′s CG is lower than the wing.
2. It produces more lift compared to mid‐ and low‐wing configurations.
3. It has a better gliding ratio.
4. The aircraft has a lower stall speed since its *C*_*L*_ is higher.
5. There is more space inside the fuselage available, and it is easy to build.

The wing dihedral and anhedral are neglected and considered to be zero for small‐scale glider design [21].

#### 2.2.2. Tail Airfoil Selection

The tail airfoil sections for both horizontal and vertical stabilizers are selected to be symmetrical airfoils to generate zero lift force at zero angle of attack. The NACA 0012 airfoil is selected. It is obvious from Figure 4 that the NACA 0012 airfoil does not generate lift during cruising flight at zero angle of attack.

Figure 4S1223 airfoil characteristics *Y* with the change in angle of attack alpha *X* at different values of Reynolds number [22]. (a) Left coefficient Cl. (b) Cl/Cd.

**Figure:**
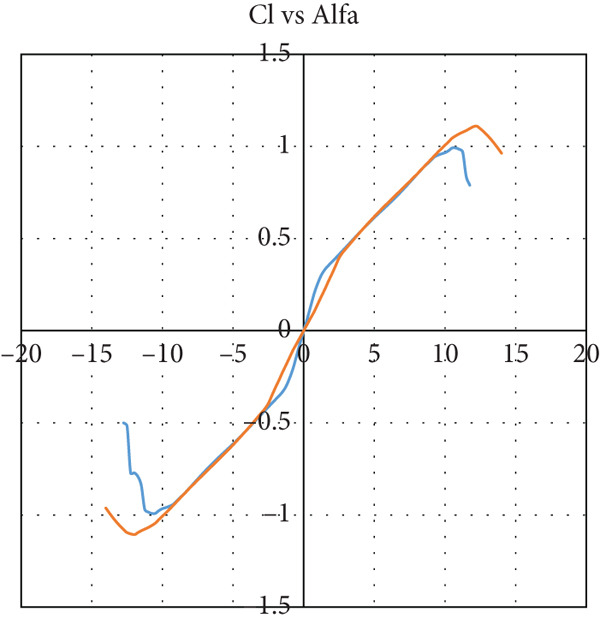
(a)

**Figure:**
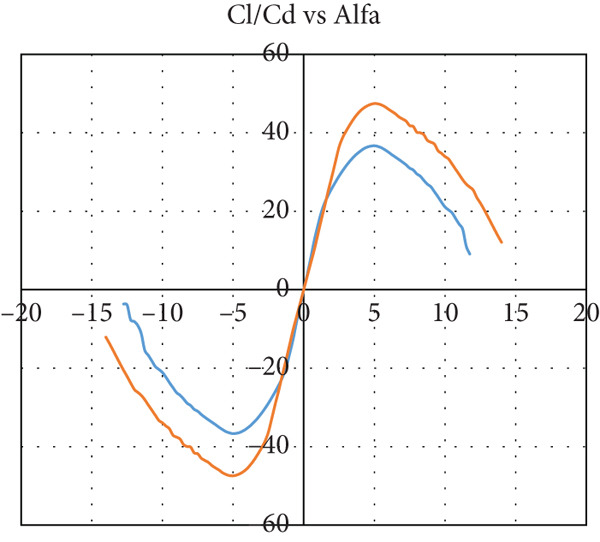
(b)

#### 2.2.3. Landing and Take‐Off

One of the most important design aspects of any aircraft is landing and take‐off. Landing is designed to be achieved by a parachute.

The parachute is considered hemispherical in shape and is designed to provide a safe landing for the glider. The terminal speed for landing when the glider hits the ground is equivalent to a free fall from a height of 1.3 m, that is, a 5 m/s terminal speed. According to the simplified design analysis presented in Appendix A, the required parachute has a diameter of 1.52 m. The total weight of the parachute system is 0.25 kg.

Glider take‐off is designed to be achieved by a helium balloon. The balloon will raise the glider to the targeted altitude of 100 m using its buoyancy force within a reasonable time. A detailed analysis for the balloon sizing is presented in Appendix B. The analysis is based on a 5 kg total weight of the glider system (glider weight, tether with conducting element, balloon weight, balloon tether weight, and parachute weight) excluding the helium weight. The balloon is assumed to be spherical in shape. Once the glider reaches the targeted altitude, the balloon will be detached and pulled down to the ground. The balloon will be deflated and helium will be collected for the next flight. The results presented in Appendix B show that the glider will, under ideal conditions, reach the target altitude in approximately 12 s. According to Table A1 and the selection made in Appendix B, the balloon tether weighs 0.5 kg/100 m.

#### 2.2.4. Tether Selection

The task of the tether is to keep the glider in place and connected to the ground station. High modulus polyethylene (HMPE) fiber from Dyneema is selected, as presented in Appendix C. The tether is equipped with a conducting element to allow power transmission to the ground station. The total weight of the tether is 1.5 kg/100 m.

### 2.3. Geometry Modeling

The actual glider model consists of a large number of assembled parts. The simulation model is built to replicate the prototype; that is, the spars and ribs are modeled. This increases the accuracy of the simulation results and simplifies the manufacturing and implementation processes. The geometric model comprises wing, body, and tail assemblies.

#### 2.3.1. The Wing

**Figure 5:**
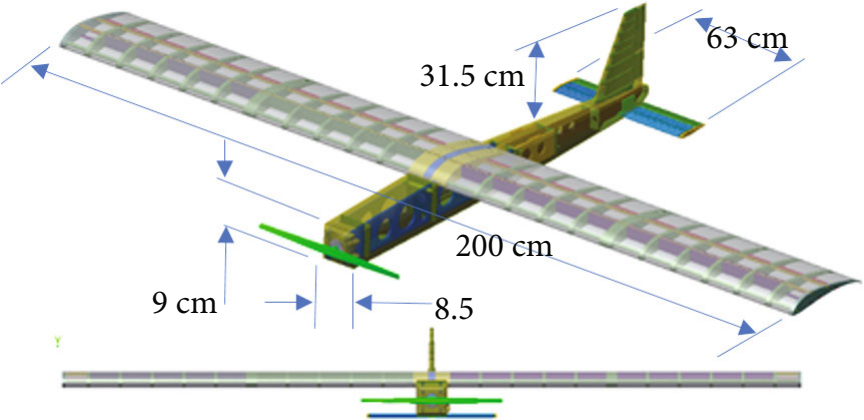
The glider CAD model.

The wing is mainly composed of ribs, spars, and an external coating. Spars are long beams that provide most of the strength and structural support for the wing. They run from the fuselage to the wing tip. Ribs form the actual shape (or camber) of the wing and are attached to the spars. Figure 5 illustrates the wing model. The wing is modeled with an initial angle of attack of 3° to increase the generated lift force.

#### 2.3.2. The Body

The function of the aircraft body is to support the wing and tail and to carry all the equipment. Therefore, it must be able to withstand the bending moments caused by weight and lift forces, as well as torsional loads. Figure 6 presents the body design, which aims to be as light, strong, and easy to manufacture as possible. It is composed of two parts: the fuselage structure and the body cover or skin. The body assembly consists of frames and spars to support and reinforce the body structure and the body cover.

Figure 6The glider body. (a) The main structure. (b) The body cover.

**Figure:**
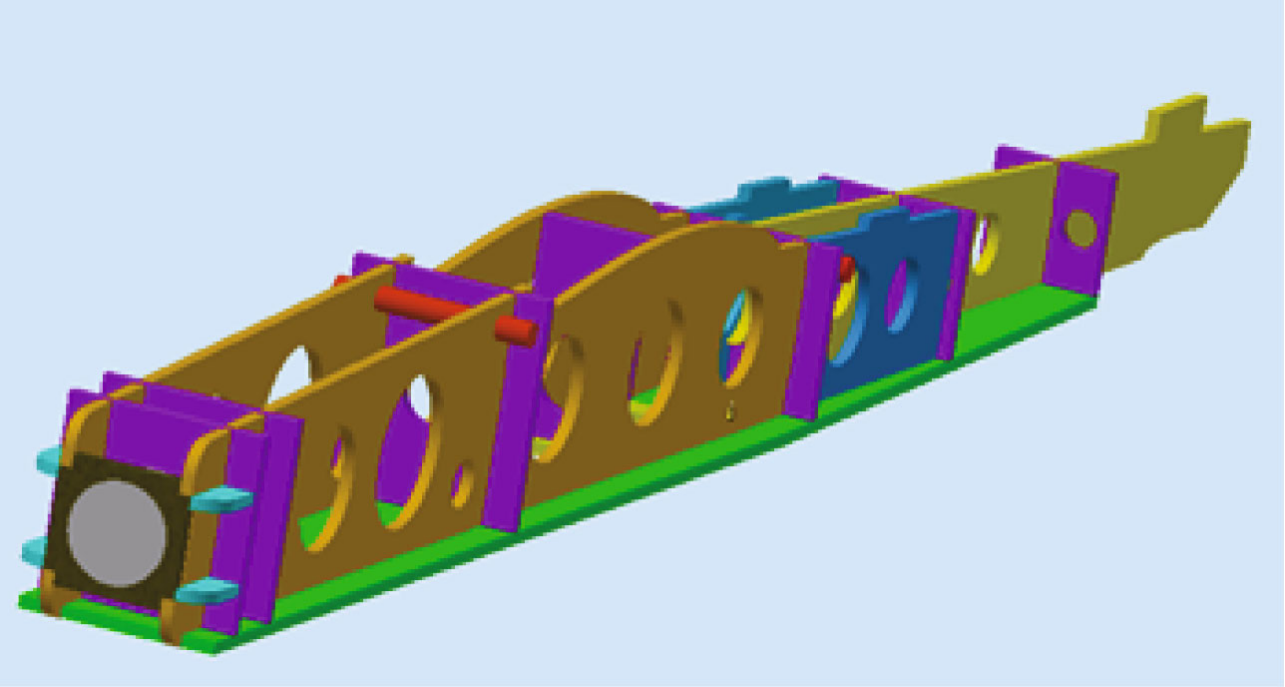
(a)

**Figure:**
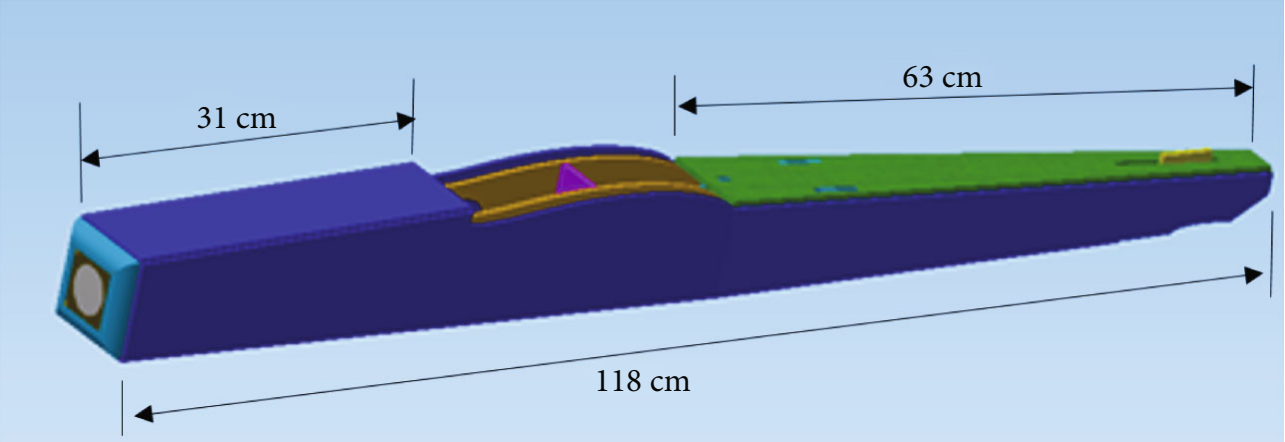
(b)

The fuselage is equipped with two service doors in the belly, as shown in Figure 7a, a generator and turbine mount in the nose, as shown in Figure 7b, and a tether mounting point, as shown in Figure 7c.

Figure 7Fuselage. (a) Service doors. (b) Generator and turbine mount. (c) The tether mounting point.

**Figure:**
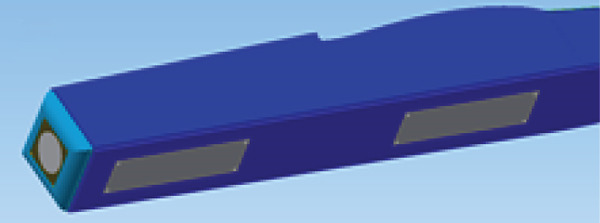
(a)

**Figure:**
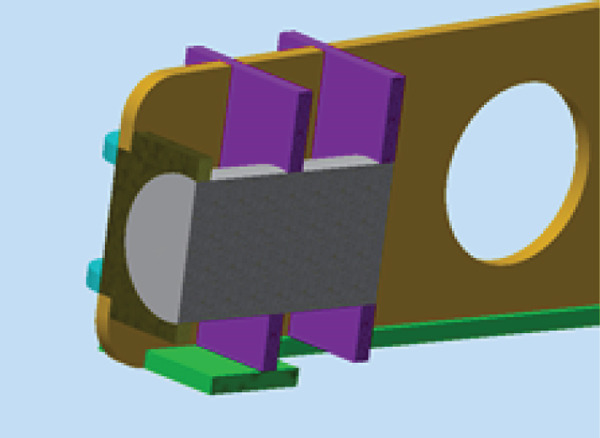
(b)

**Figure:**
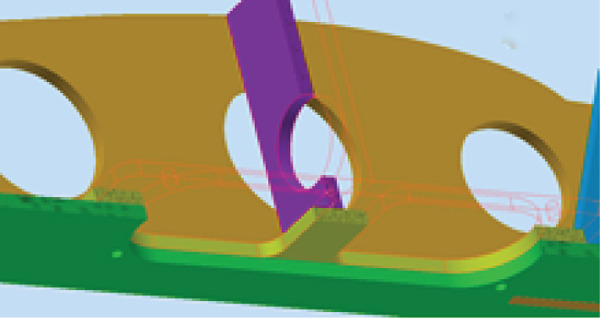
(c)

#### 2.3.3. Tail Section

The tail of an aircraft is designed to provide both stability and control for the aircraft in pitch and yaw maneuvers. The tail section consists of the vertical stabilizer that controls yaw maneuver by the rudder and the horizontal stabilizer that controls pitching maneuver by the elevator. Figure 8a presents the horizontal stabilizer and the elevator, whereas Figure 8b presents the vertical stabilizer and the rudder.

Figure 8The tail section. (a) Horizontal stabilizer and elevator. (b) Vertical stabilizer and rudder.

**Figure:**
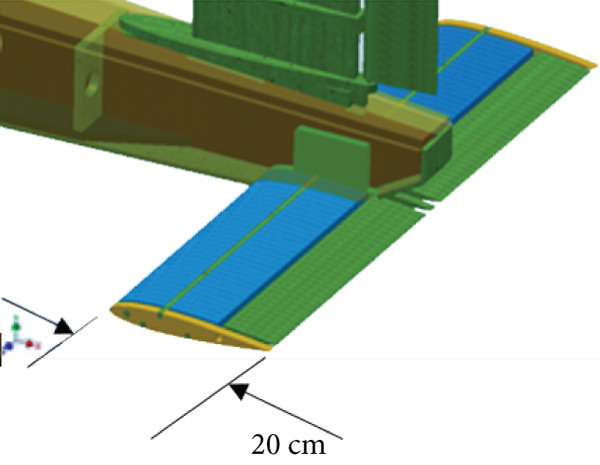
(a)

**Figure:**
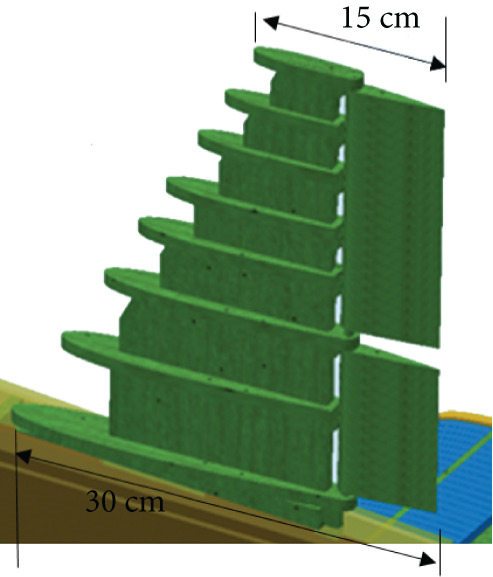
(b)

### 2.4. CFD Model

CFD analysis is conducted on the glider model. The results of air velocity, static pressure, lift and drag forces on the wing, and forces on the glider body are collected. The results are obtained for different critical flight positions, from the normal flight case to the worst scenario. XFlow software is utilized in creating the CFD.

#### 2.4.1. The Numerical Element Model

XFlow that is utilized in the aerodynamic modeling employs an octree structure to discretize the geometry and automatically adapts the resolved scales to the user′s requirements. XFlow uses a particle‐based kinetic solver and avoids the traditional meshing process. XFlow is based on the lattice Boltzmann method, a particle‐based approach that offers advantages in handling complex geometries and moving boundaries compared to traditional mesh‐based CFD methods.

To assess the quality of the simulation, users can focus on the refinement of the lattice near walls and the convergence of global quantities like lift and drag coefficients. Automatic lattice generation in XFlow minimizes user inputs, reducing time and effort in the meshing and preprocessing phase. Adaptive mesh refinement in XFlow is utilized where XFlow automatically refines the mesh near walls and areas with strong gradients to capture flow features accurately. XFlow uses a high‐fidelity wall‐modeled large eddy simulation (WMLES) approach for turbulence modeling. This approach relies on the wall‐adapting local eddy (WALE) viscosity model as its underlying LES technique, providing a consistent local eddy‐viscosity and near‐wall behavior. XFlow′s WMLES is particularly well suited for complex transient flow scenarios and moving geometries. XFlow′s turbulence modeling approach, combined with its particle‐based technology, enables accurate and efficient simulation of complex turbulent flows, including those involving moving parts, multiphase flows, and fluid‐structure interactions [24, 25].

**Figure 9:**
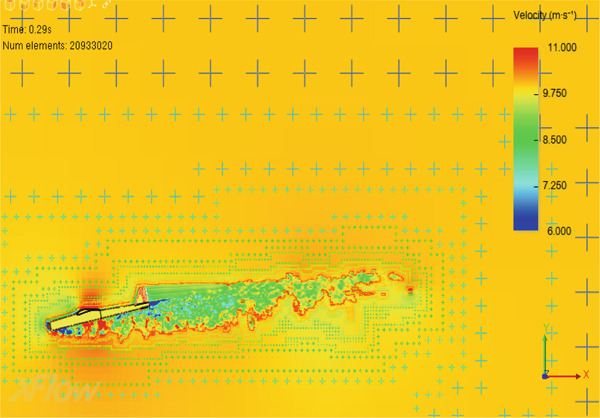
The CFD mesh.

The plus (+) signs in Figure 9 represent the numerical elements around the glider model. The small plus signs represent small numerical element sizes, whereas the larger ones represent a coarse mesh. The mesh is refined until mesh independence results are achieved, that is, a stable solution is reached. The numerical element size near the model body is 0.00625 m, while the far‐field element size is on the order of 0.2 m. The total number of numerical elements is 20,933,020 as shown in the upper left corner of Figure 9. To assure the accuracy of the finite element model, analytical analysis is carried out for the lift force generated by the wing and compared with that obtained from the CFD model. Both results are very close, with a difference within 5%.

The wind tunnel in the CFD simulation is modeled as a large space of size 20 × 7 × 7 m with air moving inside. The CFD simulation is designed to replicate the actions of an actual object in flight. A steady state must be reached within 0.2–0.3 s; otherwise, it will be considered unstable. Therefore, the focus of the simulation results will be on the first 0.5 s. Four main study cases will be conducted: cruise, nose‐up, nose‐down, and cruise with a turbine installed. The stability control system is not utilized to adjust the flight state of the aircraft, replicating the worst flying scenarios. Air properties are set to sea‐level conditions for all cases. In all cases, the model is allowed to freely rotate around its lateral axis. The following is a description of each case.

#### 2.4.2. Cruise Flight

Two cruise flight configurations were conducted: one with the center of mass forward of the aerodynamic center and another with the center of mass aft of the aerodynamic center. For both cruise cases, the climb angle is set to zero. The wing is installed on the fuselage to achieve an angle of attack of 3° at a zero climb angle.

#### 2.4.3. Nose‐Up Flight

In this case, the climb angle is 9°, resulting in a 12° angle of attack on the wing. This is the critical angle of airfoil S1223‐il (see Figure 3), near the stall point where the airfoil efficiency drops sharply. This extreme flight condition was simulated to obtain the maximum forces the model might experience.

#### 2.4.4. Nose‐Down Flight

The glider dive angle in this case is −12°, that is, the wing angle of attack is −9°. This is another extremely critical case for the glider. According to Figure 3, the glider loses its lift force and experiences a free fall.

#### 2.4.5. Cruising Fly With Turbine‐Equipped Case

The turbine is added to the model in this case to investigate its effect on the glider′s performance. As in the previous cases, the glider is allowed to freely rotate around its lateral axis.

### 2.5. Structural Model

The CFD investigation generated a huge amount of data on the forces and loads that the glider experienced during the flights. The data was utilized to simulate and analyze the structure of the glider design using FEA to determine whether it can withstand the loading or not. FEA is carried out for both the wing and the body.

The structure of the glider is made of balsa wood, as mentioned earlier. Balsa wood is widely used in R/C trainer aircraft for its light weight, low cost, and ease of shaping. It is suitable for low‐speed aircraft but needs careful handling due to its low strength. It has the lowest density among woods, the highest dielectric strength, and moderately low ductility [26]. Most of the glider structure is balsa wood, but aluminum tubes are also utilized in the wing structure. Three aluminum tubes along the wing span are added to carry the aerodynamic and structural loads. Each tube has a diameter of 10 mm and a thickness of 0.5 mm. One tube is placed near the wing leading edge, a second near the aerodynamic center, and a third near the trailing edge. The material properties used in the structural simulation are presented in Table 1.

**Table 1:** Balsa wood and aluminum mechanical specifications [26].

**Property**	**Balsa wood**	**Aluminum**
Density	130 kg/m^3^	2700 kg/m^3^
Compressive (crushing) strength	7.0 MPa	530 MPa
Elastic (Young′s, tensile) modulus	3.0 GPa	71.7 GPa
Elongation at break	1.2%	0.1%
Poisson′s ratio	0.38	0.33
Shear modulus	0.23 GPa	26.9 GPa
Ultimate tensile strength: UTS	14 MPa	324 MPa
Yield strength	—	169

**Figure 10:**
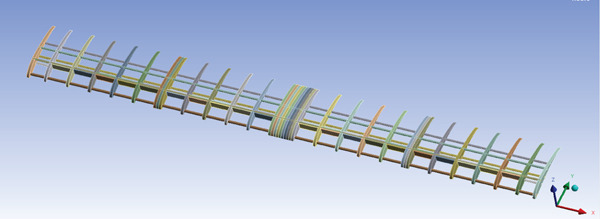
The wing structure.

**Figure 11:**
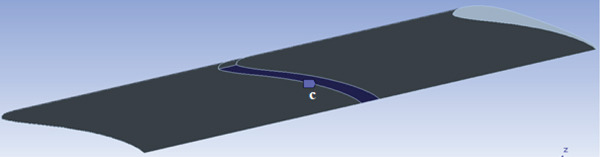
The wing support.

The wing is the most critical part of the glider that is responsible for aircraft lift and stability. The wing consists of ribs, spars, and beams that support a 1‐mm skin, as shown in Figure 10. The wing is considered to be a fixed‐supported cantilever beam. It has fixed support in the middle at point (c) where it is attached to the fuselage, as shown in Figure 11. The wing structure is meshed using solid brick elements. The mesh is refined until the change in the obtained results does not exceed 1% when the element size is 1 mm, that is, the results are mesh‐independent.

**Figure 12:**
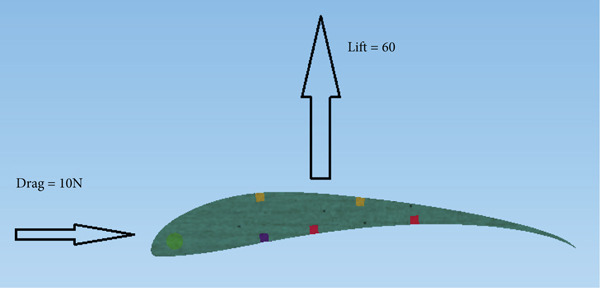
Cross‐sectional view of the load case.

The CFD analysis load results are applied to the model. Figure 12 presents the resultant lift and drag forces on the wing obtained from the CFD analysis. The loads are applied to the wing as obtained from the CFD analysis, that is, as distributed loads over the wing surface area.

## 3. Analysis

The data extracted from the CFD simulation for the glider is employed to investigate the load‐carrying capacity of the structure and ensure that it meets the targeted performance. The results show that the system can carry the structural and aerodynamic loads.

### 3.1. Numerical Calculations

To achieve the aircraft (glider) design and modeling, the predesign parameters are set as follows: the glider total weight is 3.35 kg, the wing chord length is 25 cm, and the WCL lies between 3.5 and 5.5. The results obtained for the wing span are shown in Table 2. The desired wingspan *L*_*w*_ is that which can generate the lift force *F*_*L*_ needed to carry at least a weight of 3.35 kg (i.e., 32.86 N) at a wind speed V_W of 10 m/s according to the data of S1223‐IL presented in Figure 2. The total weight estimation of 3.35 kg is shown in Appendices A, B, and C. This weight includes the glider weight of 1.6 kg, the tether with conducting element weight of 1.5 kg, and the parachute system weight of 0.25 kg.

**Table 2:** Wingspan selection.

**WCL (wing cube load)** $WCL=\frac{W_{total}}{A_{s}^{1.5}}$	**L**_**w**_ **(cm)**	**AR (aspect ratio)**	**F**_**L**_ **(N) at alpha 0**
3.5	237.37	9.50	42.73
4	217.15	8.69	39.09
4.5	200.75	8.03	36.14
5	187.14	7.49	33.69
5.5	175.62	7.03	31.61

The design target for the wing size is to be in the range of 200 cm. A WCL of 4.5 is chosen since it achieves the design target wingspan *L*_*w*_ of 200 cm for a wing chord *C*_*m*_ of 25 cm and an AR of 8. The lift force estimation is shown in Figure 33 for three values of Re number: Re = 100,000; 125,000; and 165,500. The white cells represent no‐fly states since the lift force is less than that needed to carry the load. The gray cells represent the region where the targeted performance is achieved. For a wind speed of 7.5 m/s, the targeted performance is achieved at angles of attack ranging from 8 to 11.5°. Similarly, the targeted performance is achieved for a wind speed of 10 m/s at angles of attack ranging from 2° to 11.8°. Evidently, as the wind speed increases, a smaller angle of attack is needed to reach the desired performance.

To achieve the aircraft (glider) design and modeling, the predesign parameters are set as the following: the glider total weight is 3.35 kg, the wing chord length is 25 cm, and WCL is laying between 3.5 and 5.5. The results obtained for the wing span are shown in Table 2. The desired that can generate the lift needed to carry at least to carry 3.35 kg (i.e. 32.86 N weight) at wind speed *V*_*W*_ of 10 m/sec according to the data of S1223‐il presented in Figure 2. The total weight estimation of 3.35 kg is shown in Appendices A, B, and C. This weight includes the glider weight of 1.6 kg, the tether with conducting element weight of 1.5 kg, and the parachute system of 0.25 kg.

The design target for the wing size is to be in the range of 200 cm. So WCL of 4.5 is chosen since it achieves the design target of 200 cm for a wing of 25 cm and AR is 8. The lift force estimation is as shown in Figure 33 for three values of Re number; Re = 100,000; 125,000; 165,500. The white cells represent no fly states since the lift force is less than what is needed to carry the load. The gray color cells represent the region where the targeted performance is achieved. For a wind speed of 7.5 m/s, the targeted performance is achieved when the angle of attack range is between 8° and 11.5°. Also, the targeted performance is achieved for a wind speed of 10 m/s when the angle of attack range is between 2° and 11.8°. It is obvious as the wind speed increases, a smaller angle of attack is needed to reach the sought performance.

### 3.2. Simulation Results

The simulation analysis consists of two main parts: the first is CFD for aerodynamic analysis, and the second is structural FEA. XFlow software is used in the CFD analysis, whereas the structural analysis is carried out by ANSYS software.

To ensure that accurate results are obtained for the structural model, the following procedure is applied:

• A proper finite element type that suits the model is selected. Solid elements are selected for the structural model. These elements carry shear loads, whereas tetrahedral elements are abandoned since they are considered stiff elements.
• The mesh is refined until the difference between successive iterations is less than 1%. This rules out the mesh effect.

### 3.3. CFD

Five different cases are investigated. The first case is a cruise flight with the aerodynamic center located ahead of the center of mass. The second case is also a cruise flight with the center of mass located ahead of the aerodynamic center, which is the stable arrangement for the airplane design. This arrangement is considered for all other cases. The third and fourth cases are nose‐up and nose‐down flights, respectively. The turbine is added to the airplane in the last case to investigate its effect on the flight performance.

#### 3.3.1. AC Upfront Cruise Flight

**Figure 13:**
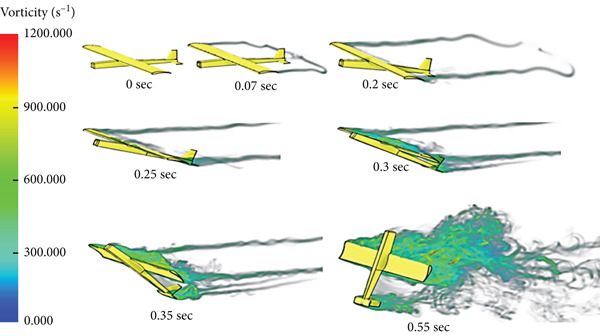
Vorticity illustration for AC upfront cruise.

In this case, the aerodynamic center is placed ahead of the CG of the glider. Due to this configuration, the glider tends to flip over during the flight, that is, a stall takes place. Figure 13 shows the case simulation at different time steps.

#### 3.3.2. Normal Cruising Flight

**Figure 14:**
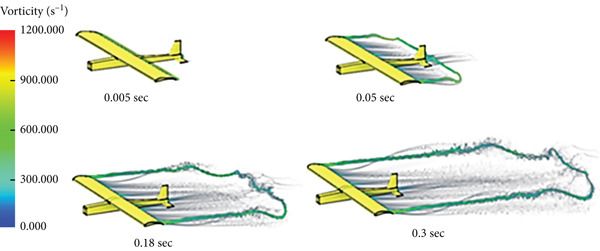
Vorticity illustration for normal cruise flight.

The glider is modified so that the CG is moved ahead of the aerodynamic center to achieve stability. Figure 14 shows different stages of the CFD simulation. The flight is stable and smooth.

**Figure 15:**
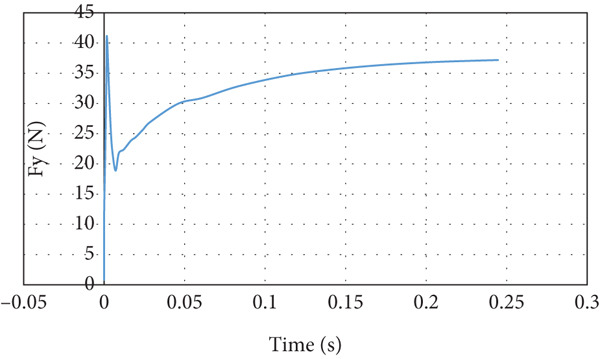
Lift force curve for normal cruise flight.

**Figure 16:**
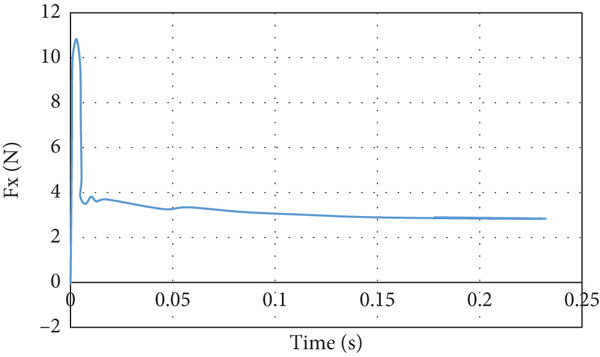
Drag force curve for normal cruise flight.

Figures 15 and 16 present the behavior of the resultant lift and drag forces acting on the aircraft, respectively. The lift force, as can be seen in Figure 15, goes up and down within 0.01 s as the air strikes the aircraft and then starts to converge smoothly to 39.1 N. The reason for the sharp jump in the lift force at the beginning is to overcome the high drag force that reaches its peak value when the air starts striking the aircraft. The drag force overshoots to approximately four times the steady‐state drag force of 2.87 N, as illustrated in Figure 16.

**Figure 18:**
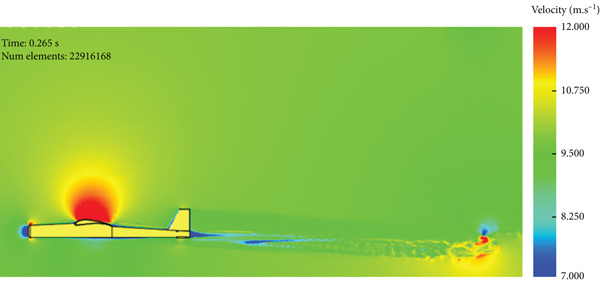
Air velocity distribution around the glider for normal cruise flight.

Figure 17a,b presents the static air pressure and airspeed distributions. It can be observed that static pressure is lowest at the upper surface of the wing where the highest airspeed occurs. The airspeed at the upper surface of the wing reaches 12 m/s. The lowest static air pressure developed at the upper surface is −80 Pa, while the pressure at the bottom surface of the wing is 40 Pa. The wing surface′s lowest pressure occurs at the first one‐third of the wing chord. Figure 18 shows the velocity distribution in the space around the glider. It is obvious from Figure 18 that the maximum airspeed occurs over the wing.

Figure 17Normal cruise flight. (a) Static pressure distribution. (b) Velocity distribution.

**Figure:**
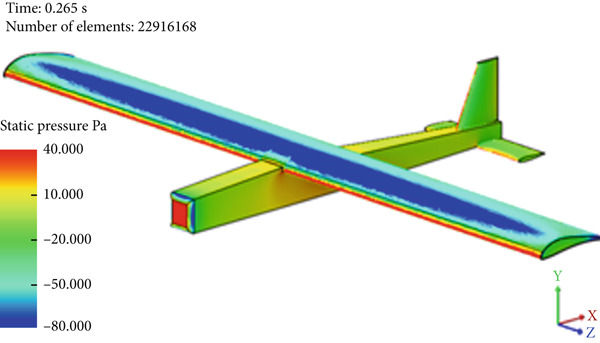
(a)

**Figure:**
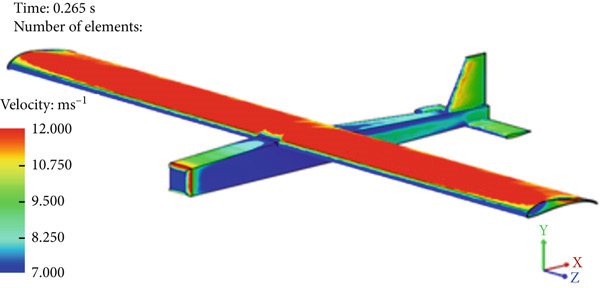
(b)

#### 3.3.3. Nose‐Up Flight

**Figure 19:**
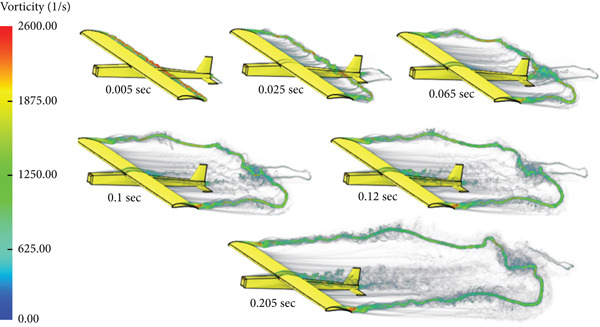
Vorticity of nose‐up flight.

The aircraft in this case has an initial climb angle of 9°, that is, an angle of attack of 12°. Figure 19 shows the flight simulation for the nose‐up flight. Compared to the normal cruise case, a large wake and disturbance can be observed at the rear of the glider due to the sharp climb and high angle of attack.

**Figure 20:**
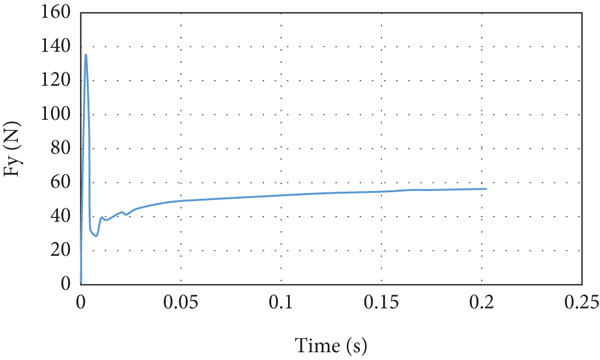
Lift force curve for nose‐up flight.

**Figure 21:**
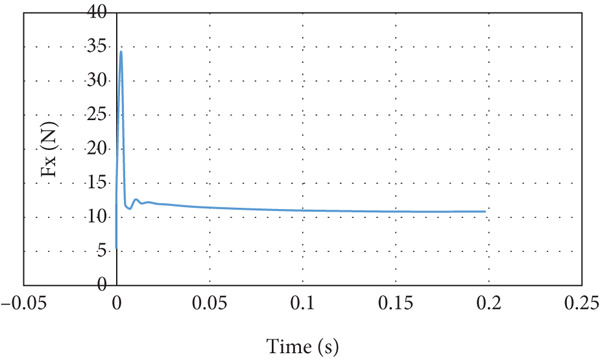
Drag force curve for nose‐up flight.

**Figure 22:**
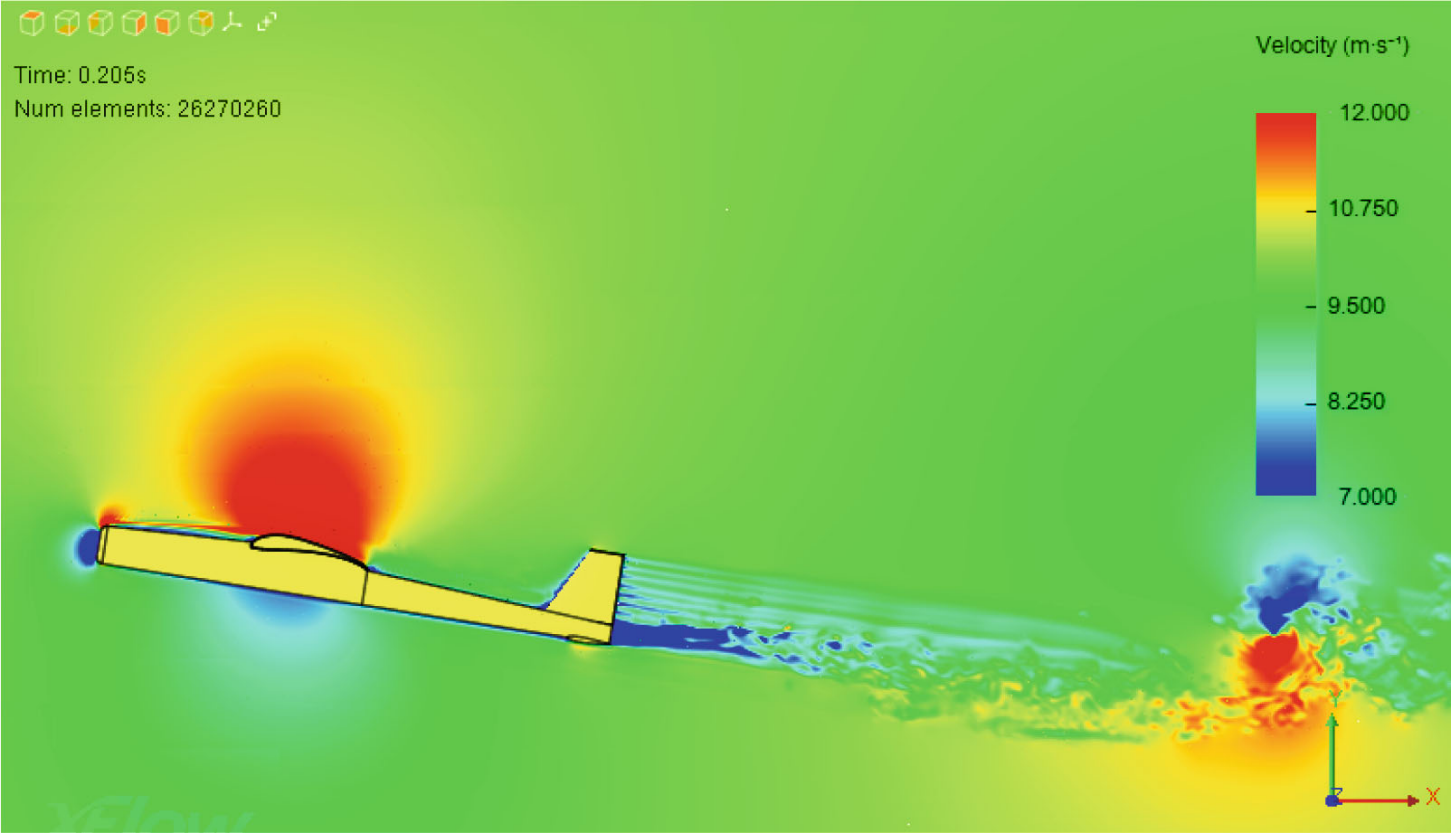
Air speed distribution around the glider for nose‐up flight.

The lift force overshoots to a maximum of 135 N within 0.01 s, as in the previous case, and reaches the steady‐state level of 60 N in 0.2 s, as shown in Figure 20. The drag force also overshoots to a maximum of 35 N within 0.01 s and converges to 6.7 N in 0.2 s, as can be seen in Figure 21. The airspeed distribution in the space around the glider is presented in Figure 22. The envelope of the high‐speed distribution is larger than that in the cruise case, and the wake behind the aircraft is more pronounced.

The simulation shows a broadening of the negative pressure and high‐speed areas on the top surface of the wing, as illustrated in Figure 23. This explains the increase in disturbance in the air, especially over the middle region and the wing tips.

Figure 23Nose‐up flight. (a) Static pressure distribution. (b) Velocity distribution.

**Figure:**
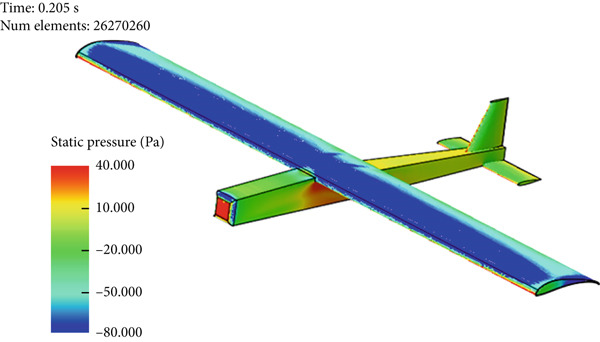
(a)

**Figure:**
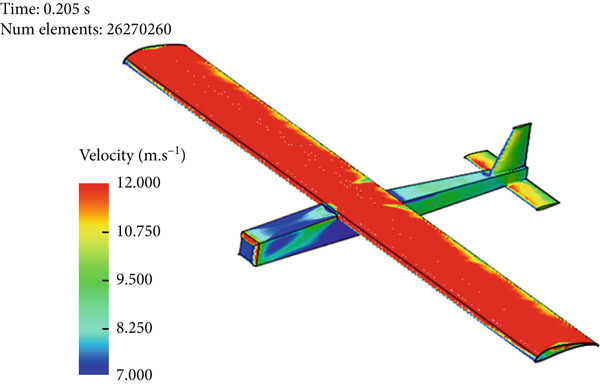
(b)

#### 3.3.4. Nose‐Down Flight

**Figure 24:**
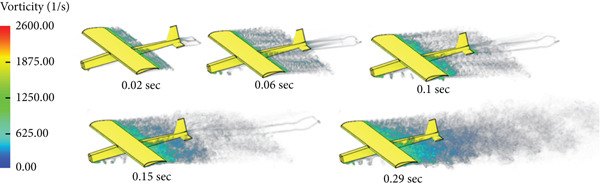
Vorticity of nose‐down flight.

The simulation for nose‐down flight is presented in Figure 24. A huge disturbance in the air can be easily noticed at the nose and the wing trailing edge. The diving angle in this case is −12°, that is, the angle of attack is −9°.

**Figure 25:**
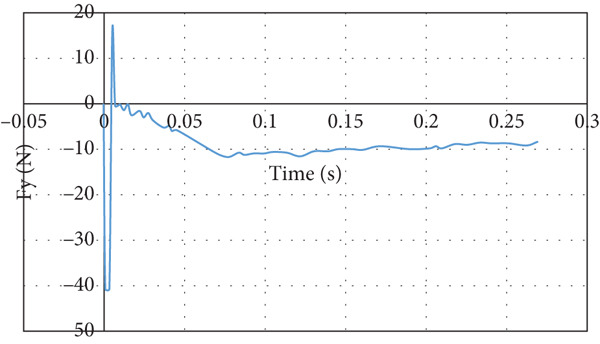
Lift force curve for nose‐down flight.

**Figure 26:**
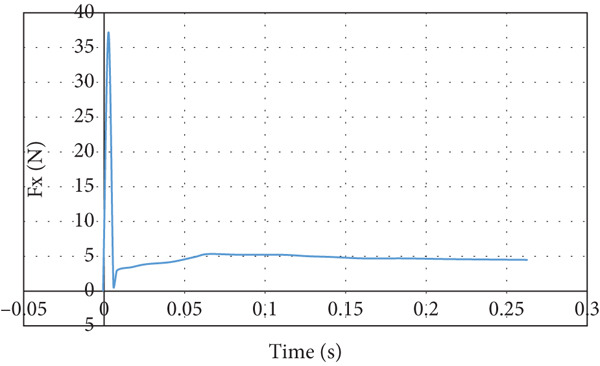
Drag force curve nose‐down flight.

The lift force behavior is shown in Figure 25. The lift force overshoots initially in the negative direction and converges to a negative lift force that is acting downward on the wing. The downward lift force converges to −10 N. The drag force overshoot is eight times the converging value of 4.55 N, as illustrated in Figure 26.

In this case, the positive pressure starts to build up on the upper surface of the wing, especially on the leading edge and the first one‐third of the wing camber, as can be seen in Figure 27a. Accordingly, the air speed decreases sharply at the wing leading edge, as illustrated in Figure 27b, which leads to the increase in the static pressure at the top surface of the wing.

Figure 27Nose‐down flight. (a) Static pressure distribution. (b) Velocity distribution on the upper and lower surfaces of the wing.

**Figure:**
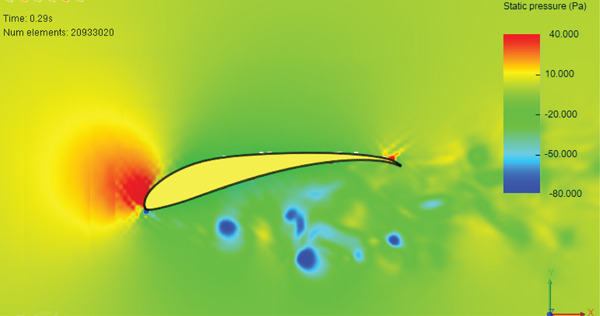
(a)

**Figure:**
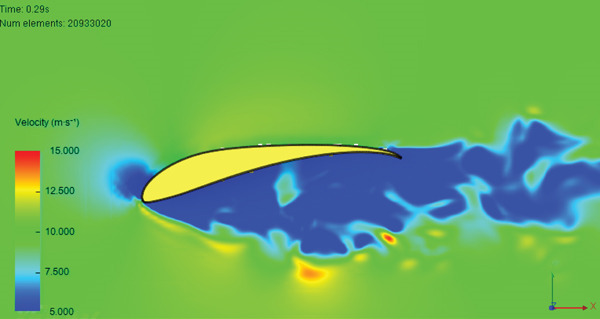
(b)

#### 3.3.5. Cruise Flight With Turbine Installed

**Figure 28:**
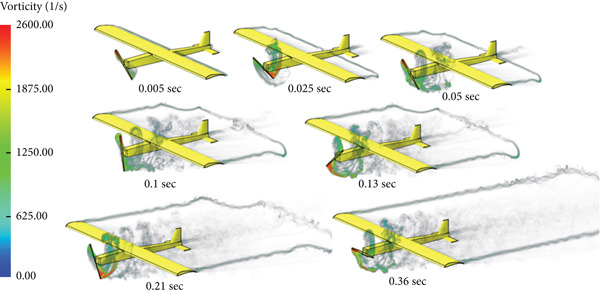
Vorticity for cruise flight with turbine installed.

The simulation in Figure 28 shows that the rotation of the turbine mounted at the aircraft nose disturbs the airflow and affects the quality of the air delivered to the wing part shielded by the swept area of the turbine blade.

**Figure 29:**
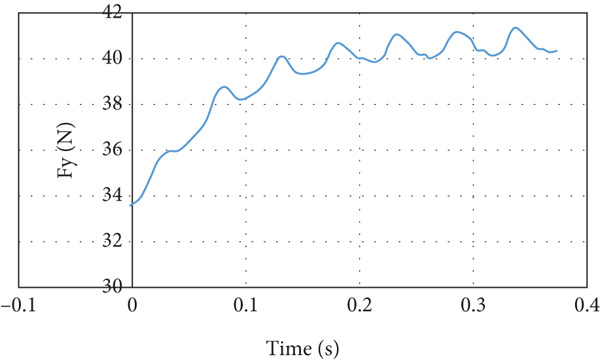
Lift force curve for cruise flight with turbine installed.

**Figure 30:**
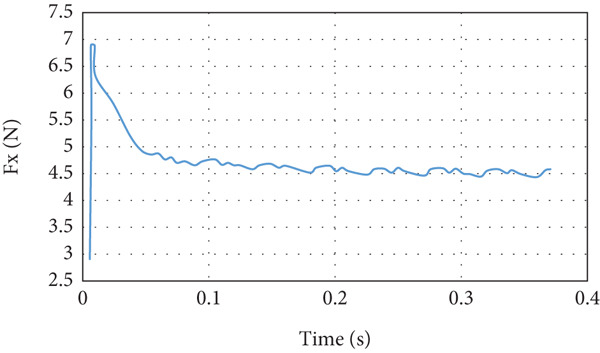
Drag force curve for cruise flight with turbine installed.

The lift force builds up smoothly initially and then it starts to fluctuate due to the rotation effect of the turbine blade, as can be seen in Figure 29. The fluctuation range is small; it is from 40 to 41.25 N at the steady state, that is, within ±3%. It can be noticed that there is no overshoot in the lift force. The overshoot in the drag force is very small compared to the other cases, as demonstrated in Figure 30. The drag force is affected by the turbine blade rotation, but the fluctuation is within ±0.1 N.

A summary of the CFD results is presented in Table 3. Four main cases are shown in the table: cruise case with the forward CG (Case 2), nose‐up position (Case 3), nose‐down position (Case 4), and cruise with the turbine mounted on the aircraft body (Case 5). The table demonstrates the differences between the cases for the lift force, lift force overshoot, drag force, drag force overshoot, angle of attack, and turbine presence. The comparison is carried out at an air speed of 10 m/s.

**Table 3:** Simulation summary of the lift‐drag analysis.

**Case #**	**2**	**3**	**4**	**5**
Lift (N)	39.1	59.8	‐9	40
Lift overshoot (N)	43	135	15	—
Drag (N)	2.87	6.7	4.5	4.5
Drag overshoot (N)	12	40	38	10
Angle of attack (degree)	3	12	−9	3
With turbine	No	No	No	Yes

### 3.4. Structural Simulation Results

The wing was tested under a 60‐N lift force and a 10‐N drag force applied as a resultant distributed load over the wing surface, as shown in Figure 12. The design results depend on the balsa wood properties listed in Table 1, which has the least strength among the materials in the structure.

**Figure 31:**
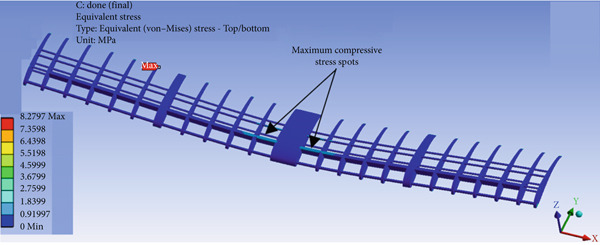
FEA stress analysis of the wing structure.

**Figure 32:**
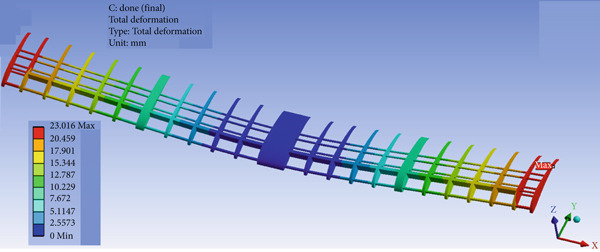
FEA deflection for the wing.

The wing design presented in Figure 10 shows promising performance, as demonstrated in Figures 31 and 32. The maximum compressive (crushing) stress was 0.91 MPa at the top surface of the balsa wood spars, and the maximum tensile stress was 8.27 MPa at the bottom surface opposite the maximum compression spot, as illustrated in Figure 31. The compression factor of safety is 7, and the tensile factor of safety is 1.7. The design also shows acceptable deflection: 0 mm at the root (where the wing is attached to the fuselage) and 23 mm at the tip of the wing, as presented in Figure 32.

## 4. Discussion

The glider tended to flip over during the cruise flying in the first case of CFD simulation, neglecting the control surfaces that can correct the situation, due to misalignment of loads. In this case, the CG is behind the center of aerodynamics (CA); this condition seizes the glider′s stability and the ability to sustain flying and causes a stall at the end. The lift force generates a moment at the center of mass that causes the airplane to lose its stability and flip over.

In the second case and thereafter, the CG is proceeding CA. The simulation of this cruise flying shows that the generated lift force is 39.09 N and the drag force is 2.87 N. The developed lift force is acceptable since it exceeds the needed force for carrying the load. Overshoot is noticed in lift and drag forces presented in Figures 15 and 16, respectively. The overshoot usually occurs due to flow acceleration [27]. The airspeed in the wind tunnel simulation is accelerated from 0 to 10 m/s. Such a situation simulates the worst case scenario when the aircraft experiences wind gusts. As shown in Table 3 and Figures 15 and 16, the lift force overshoot is 135 N and the drag force overshoot is 40 N.

Glider in Case 3 is in a nose‐up flying position. The incline angle of the aircraft is set to 9°, so the angle of attack is 12° since the wing is installed at an initial angle of attack of 3°. The steady‐state lift force increases to 60 N compared to 39 N in the cruising case, as illustrated in Figure 21. The steady‐state drag force increased as well to a maximum of 6.7 N compared to 2.9 N in cruising, as presented in Figure 22. This is due to the increase in the projected frontal area that faces the wind, which leads to an increase in the wake and the disturbance of air at the middle of the wing, at the top of the fuselage, and at the wing tips, as shown in Figure 19. The high air speed envelope over the wingspan is very large compared to the other cases (Figure 18), as demonstrated by Figure 23, due to the high angle of attack. The static pressure over the wing area is very low and widely spread over the wing top surface compared to the other cases (see Figure 17), as presented in Figure 23a. This is explained by the broadening of the high speed at the wing top surface area, as illustrated in Figure 23b.

**Figure 33:**
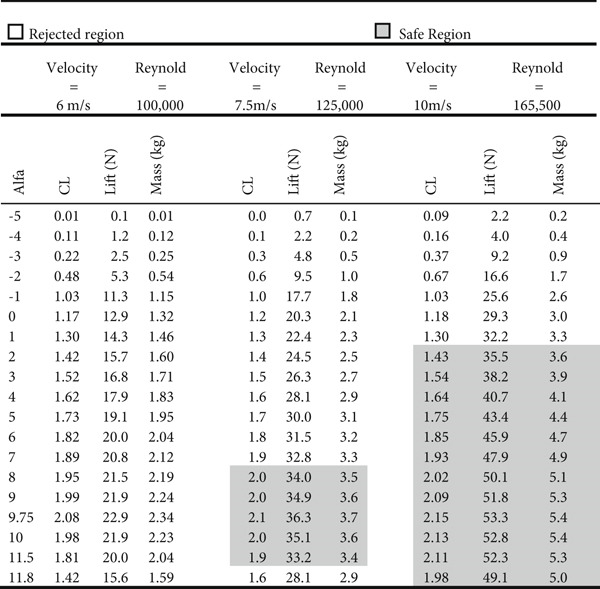
Lift force estimation.

The CFD simulation of nose‐down flying is illustrated in Figure 24. The climb angle is set to −12 so the angle of attack is −9°. This causes the lift force to be inverted in the direction due to the built‐up of positive pressure on the top surface of the wing, mainly at the leading edge, as demonstrated in Figure 27a. This is clarified by the high reduction of airspeed at the upper surface of the wing, as presented in Figure 27b. This causes the glider to dive due to the negative lift force of 8.97 N that is developed at the wing, as illustrated in Figure 25. The overshoot in the lift force of 15 N is small compared to the other cases due to the high turbulence that took place. Due to the high turbulence, the overshoot in the drag force is relatively large, as illustrated in Figure 26. According to Figure 33, this limits the working region to be not lower than a 2° angle of attack at a wind speed of 10 m/s.

The last case is cruise flight with the wind turbine installed at the nose of the glider, as presented in Figure 28. The effect of the wind turbine appears in the lift force and drag forces as fluctuations, as illustrated by Figures 29 and 30, respectively. This affects the quality of the wind delivered to the wing. The area of the wing that lies in the shadow of the wind turbine blade′s swept area experiences more turbulence, as can be seen in Figure 28. This turbulence eliminates the overshoot in the lift force and reduces the overshoot in the drag force. However, both lift and drag forces experience fluctuations of +/−1 N. This amount of fluctuation, +/−2.5% in the force, is within design‐acceptable limits. The rotation of the wind turbine mixes the air flow before it reaches the wing, which leads to a dramatic reduction in the overshooting of both lift and drag forces. Since the turbine is mounted at the aircraft nose all the time, the overshoot experienced by the lift force at any angle of attack is going to be eliminated. So, the design is reaching the steady‐state situation when the lift force matches the glider system weight.

The maximum lift force that can be experienced by the aircraft according to the CFD simulation is 60 N. So the structural analysis is carried out by copying the distributed loads of both lift and drag forces that are developed over the wing as a result of the CFD simulation analysis. Three different structural designs for the aircraft are developed, but the successful one is being presented here. The wing is constructed from three balsa wood spars and many ribs, along with three aluminum tubes. Table 1 shows that balsa wood has the least strength, so the failure criterion is mainly considering balsa wood strength as a reference. Figure 31 shows that the maximum Von Mises stress takes place at the wing root, where the maximum bending moment is experienced. Since the lift force is acting upward on the wing, the maximum compression stress takes place at the upper surface of the spars at the root region, whereas the maximum tension stress is experienced by the same spars but at the bottom surface. The maximum load in the cruise case with the turbine installed is approximately 39 N and in the nose‐up case is approximately 60 N. For both cases, the structure is safe by ignoring the overshoot since the turbulence developed by the wind turbine blade eliminates such overshoot behavior.

The stress in the rest of the aircraft body is less than that developed at the wing and at the joint between the wing and the fuselage. The joint is supported by a relatively large piece of solid balsa wood to ensure its capacity to carry the stresses. The deformation at the wing tips is 23 mm, which is acceptable from a design point of view for a span of 1000 mm on each side.

The landing is going to be accomplished by a parachute that will be installed in the aircraft according to analysis carried out in Appendix A. This parachute opens whenever landing is needed either due to an emergency or due to regular grounding. Take‐off is going to be accomplished by a helium balloon that is sized in Appendix B.

## 5. Conclusion

The following can be concluded from this research:

• The glider showed a good ability to do its job in the Fly‐Gen AWES.
• Aeronautically, the glider shows good performance in different flight possible situations and achieved the desired design criteria to fly safely at relatively low wind speed.
• Structural‐wise, the design supports the aerodynamic functions; it is lightweight and strong enough to withstand the loads experienced by the aircraft.
• This work provides a guideline for simplified design for flying airborne wind turbine.

## 6. Future Work

In the future, the design will be implemented and tested in an open wind tunnel. Also, take‐off landing gear will be designed and constructed to serve for take‐off and landing.

## Conflicts of Interest

The authors declare no conflicts of interest.

## Funding

No funding was received for this manuscript.

## Figures and Tables

**Figure A1:**
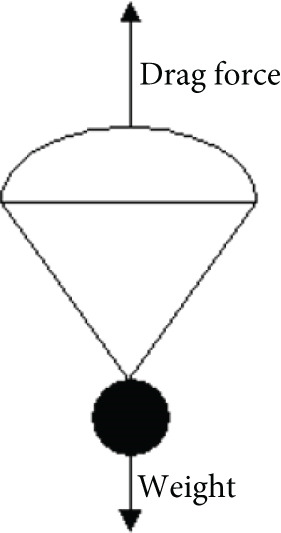
Schematic diagram for the parachute [28].

**Figure A2:**
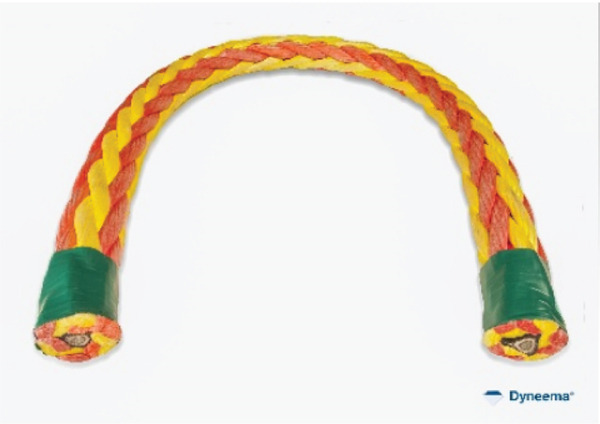
Picture of Dyneema HMPE tether [23].

## Data Availability

All data of this work are included in this manuscript.

## Appendix A: Parachute Design Analysis

This section presents simplified design analysis for the parachute illustrated in Figure A1 that is employed in landing the glider. The following assumptions are considered: the drag coefficient of the parachute is 1.75 [29]; the air density is 1.225 kg/m^3^ (International Standard Atmosphere at 101.325 kPa [abs] and 15°C, at sea level).

The descent rate of the glider and parachute system depends on how robust the glider is, on the landing surface (concrete or grass), the wind velocity, and the altitude of the glider at the time its parachute deploys (the stronger the wind and the higher the altitude, the farther the glider can deviate from the launch site). There are several other factors like the surface area of the glider and its drag. A more robust glider can descend faster than a fragile one. Hitting the grassy surface is definitely better for the glider than hitting concrete. Dropping the glider model from a height of 0.6 m is equivalent to a landing velocity of 3.5 m/s. If it is dropped from a height of 1 m, the equivalent landing velocity is 4.5 m/s. The landing velocity of 5 m/s is equivalent to a free fall from 1.27 meters. Considering all that is said, it concludes that the best descent rate is between 3.5 and 5 m/s. For the current study, a 5 m/s terminal velocity is adopted.

To size the parachute, it is necessary to find the diameter of the parachute that brings the glider down with the descent rate at which the glider does not break when it gently hits the ground. During the descent of the model just after the deployment of the parachute, two forces are acting on the glider and its parachute. Gravity accelerates the parachute and the glider toward the ground, and the drag force of the parachute pulls it in the opposite direction and slows down the descent velocity. After some time, the descent velocity will stabilize, and the gravity force *F*_*g*_ will reach equilibrium with the drag force generated by the parachute *F*_*d*_ according to Equation (A.1):

(A.1)
Fd=Fg

The force from gravity is estimated from Equation (A.2) based on the total mass of the glider system:

(A.2)
Fd=mg

where *m* is the descending glider total mass and *g* is the acceleration of gravity 9.8 m/s^2^.

The drag force generated by the parachute is calculated according to Equation (A.3):

(A.3)
Fd=12/ ρ vd2 S Cd

where *F*_*d*_ is the drag force, *ρ* is the density of air, *v*_*d*_ is the descent velocity, *S* is the area of the parachute, and *C*_*d*_ is the drag coefficient.

When the glider descends at terminal velocity, the drag force *F*_*d*_ equals the force of gravity *F*_*g*_ according to Equations (A.2) and (A.3):

12/ ρ vd2 Sp Cd=mg

From this formula, the parachute area *S*_*p*_ is determined as

(A.4)
Sp=2mg/v2 ρ Cd

Parachute shape used to recover the glider is assumed to be hemisphere in shape, that is, it has a circular projected area. For a circle, its diameter *d* is determined from its projected circular area *S*_*p*_ according to Equation (A.5):

(A.5)
d=4 Sp/π

The total mass considered in the parachute design analysis is 5 kg (see Appendix B for more details). Whereas the mass of the glider is 1.6 kg, the tether is1.5 kg and the parachute system mass as estimated below is 0.25 kg. The extra mass of 1.65 kg is a factor of safety in designing the parachute. The drag coefficient as mentioned earlier is 1.75, the air density is 1.225 kg/m^3^, and the gravity is 9.81 m/s^2^. Using the above equations, a reference area *S*_*p*_ obtained is 1.83 m^2^, so the parachute diameter is 1.52 m. This is achieved by assuming terminal speed of 5 m/s which corresponds to a free fall of the glider from a height of 1.27 m [30].

Parachute material is selected to be nylon that is often used for fabrication of parachutes. The density of the nylon utilized in the parachute fabrication is 35 g/m^2^. The surface area of hemisphere parachute is *A* = 4 *π* *r* ^2^/2; *A* = 3.63 m^2^. So the total mass of the parachute hemisphere is 0.127 kg. Considering the parachute strings mass, the total mass of the parachute system is assumed 0.25 kg [31].

## Appendix B: Balloon Sizing

The force balance Equation (B.1) is the difference between the buoyancy force and the total weight:

(B.1)
∑Fy=FB−FW=0

For a total mass of 5 kg, the total weight is *F*_*W*_ = 5 *k* *g*∗9.81 *m*/*s* ^2^ = 49.05 *N*,

 FB−49.05049.05=⟶FB=N

The buoyancy for  *F*_*B*_ can be estimated using Equation (B.2):

(B.2)
 FB=VBalloon∗gρAir−ρHelium

where *V*_*B* *a* *l* *l* *o* *o* *n*_ is the balloon volume, *ρ*_*A* *i* *r*_ is the air density, *ρ*_*H* *e* *l* *i* *u* *m*_ is the helium density, and *g* is the gravitational acceleration.

The balloon is assumed spherical in shape, so the radius of the balloon according to Equation (B.2) is evaluated as follows:

49.059.81 N=43/∗π∗R3∗m/s21.2550.164 kg/m3− kg/m3

The radius of the spherical balloon is *R* = 1.04 m. To estimate the fabric weight needed for constructing the balloon, the surface area is calculated as the following:

Surface area=413.59∗π∗R2= m2

Material selected for the balloon is Icarex fabric, which is also known as PC‐31 (Polycarbonate‐31gram/yd^2^) [32].

The density per unit area for PC‐31 is 0.03707 kg/m^2^. The mass of the helium and the balloon shell is estimated according to Equations (B.3) and (B.4), respectively:

(B.3)
MHelium=Vballoon∗ρheliumMHelium=43/∗π∗1.04 m3∗0.1640.773 kg/m3= kg

(B.4)
Mballoon shell=fabric density∗balloon surface area=0.0370713.590.50378 kg/m2∗ m2= kg

The total mass *M*_*T* *o* *t* *a* *l*_ includes (except the helium mass): glider mass *M*_*G* *l* *i* *d* *e* *r*_, tether mass with the conducting element *M*_*T* *e* *t* *h* *e* *r*_ (see Appendix C for more details), parachute system mass *M*_*P* *a* *r* *a* *c* *h* *u* *t* *e*_ (see Appendix A), balloon shell mass *M*_*B* *a* *l* *l* *o* *o* *n* *s* *h* *e* *l* *l*_, and balloon tether mass *M*_*t* *e* *t* *h* *e* *r* *for* *b* *a* *l* *l* *o* *o* *n*_. The helium mass is excluded since it is already considered in the calculations of net buoyancy force*F*_*y*_. The total mass is evaluated according to Equation (B5):

(B.5)
MTotal=MGlider+MTether+MParachut+MBalloon shell+Mtether for balloon

The balloon tether is selected SK70 DSM Dyneema of diameter 2.55 mm and mass of 0.5 kg/100 m. So the mass of the balloon tether is considered to be 0.5 kg [23]. The task of this tether is just to pull down the balloon to the ground when the glider reaches 100 m altitude above the ground. The total mass according to Equation (B.5) is

MTotal=1.61.50.250.500.5 kg+ kg+ kg+ kg+ kgMTotal=4.35 kg

The design is made for a total mass of 5 kg and the calculated is 4.35, so there is a 0.65 kg force extra to allow proper ascending speed. The extra buoyancy force devoted for speeding the rising of the balloon is estimated using Equation (B.6):

(B.6)
Force=mass∗g0.659.816.38 kg∗m/s2= N

The time elapsed *t* for the glider to reach a height of 100 m can be calculated by utilizing Equation (B.7) as follows:

(B.7)
s=−12/∗a∗t2

where *s* is the rising height and *a* is the rising acceleration.

The rising acceleration *a* is calculated based on the extra buoyancy force according to Equation (B.8):

(B.8)
Force=mass∗a⟶a=force/massa=−6.384.351.46 N/ kg=− m/s2

So the time needed to reach 100‐m height according to Equation (B.7) is estimated as the following:

1001.4611.7 m=12/∗ m/s2∗t2⟶t= s

## Appendix C: Tether Selection

Tether is needed for keeping the glider in place and for electric power conduction. High modulus polyethylene (HMPE) fiber from Dyneema illustrated in Figure A2 is selected. This tether has an electric power conduction element to transfer power to the ground station. For an altitude of 100 m, the mass of the tether, 3.4 mm in diameter according to the specification in Table A1 is 0.8 kg/100 m, with a copper core of 0.7 kg/100 m for 1 mm in diameter. The tether has a breaking load of 1300 daN, that is, it can withstand a load of up to 13 kN. The total mass of the tether with the conducting element is 1.5 kg/100 m [23].

**Table A1:** HMPE SK75 fiber tether breaking strength and mass per 100‐m length [23].

**Tethers with SK75 fiber made by DSM Dyneema**				
**Nominal diameter Ø in mm**	**“Worked in” Ø mm**	**Tether weight kg/100 m**	**Fiber weight kg/100 m** ^**a**^	**Mean breaking load daN**
1.7	1.4	0.3	0.3	350
2.55	2.2	0.5	0.5	710
3.4	2.9	0.8	0.7	1300
4.25	3.6	1.4	1.3	2400
5.1	4.3	2	1.8	2700
6.8	5.8	3.5	3.2	5500
8.5	7.2	5	4.5	9000
10.2	8.7	8.5	7.7	12,000
11.9	10.1	10.5	9.5	14,500
13.6	11.6	12.7	11.4	19,000
15.3	13	16.5	14.9	24,000
17	14.5	20	18	29,000
18.7	15.9	24.3	21.9	35,000
20.4	17.3	28.5	25.7	41,000
22.1	18.8	33	29.7	47,000
23.8	20.2	39	35.1	55,000
25.5	21.7	45.6	41	65,000
27.2	23.1	50.7	45.6	72,000
28.9	24.6	55.7	50.1	81,000
30.6	26	60.8	54.7	88,000

^a^Weight without coating for creep calculation.
